# A single domain antibody-based Luminex assay for the detection of SARS-CoV-2 in clinical samples

**DOI:** 10.3389/fimmu.2024.1446095

**Published:** 2024-08-13

**Authors:** Ellen R. Goldman, Victor A. Sugiharto, Lisa C. Shriver-Lake, Andrew M. Garcia, Shuenn-Jue Wu, Sarah A. Jenkins, Hua-Wei Chen

**Affiliations:** ^1^ Center for Biomolecular Science and Engineering, US Naval Research Laboratory, Washington, DC, United States; ^2^ Diagnostic and Surveillance Department, Naval Medical Research Command, Silver Spring, MD, United States; ^3^ Henry M. Jackson Foundation, Bethesda, MD, United States; ^4^ Leidos Inc., Reston, VA, United States

**Keywords:** single domain antibody, nanobody, SARS-CoV-2, immunoassay, nucleocapsid, Luminex, MagPlex

## Abstract

Within the past decade, single domain antibodies (sdAbs) have been recognized as unique affinity binding reagents that can be tailored for performance in a variety of immunoassay formats. Luminex MagPlex color-coded magnetic microspheres provide a high-throughput platform that enables multiplexed immunoassays. We developed a MagPlex bead-based assay for the detection of SARS-CoV-2, using sdAbs against SARS-CoV-2 nucleocapsid (N) protein in which we engineered the sdAb capture reagents to orient them on the beads. The oriented sdAbs provided an increase in sensitivity over randomly oriented sdAbs for samples of N diluted in buffer, which also translated into better detection of SARS-CoV-2 in clinical samples. We assessed the specificity of the assay by examining seasonal coronavirus clinical samples. In summary, we provide a proof-of-concept that a bead-based assay using sdAbs to detect SARS-CoV-2 is feasible and future research combining it with other sdAb-coated beads that can detect other viruses may provide a useful diagnostic tool.

## 1 Introduction

Single domain antibodies (sdAbs), also known as nanobodies or VHHs, are the recombinantly expressed variable domains from the unique heavy-chain-only antibodies found in camelids such as llamas (1). SdAbs have shown their potential value in therapeutic, diagnostic, detection, and biotechnology applications (2–5). Although at about 15 kDa they are about a tenth the size of conventional antibodies, sdAbs show the same excellent affinity and specificity as traditional monoclonal antibodies. Advantages of sdAbs over conventional antibodies include their ability to be tailored for specific applications and their inherent stability with many able to re-fold and function after denaturation (6, 7). In addition, sdAbs are typically soluble in bacterial expression and straightforward to modify or engineer. Importantly, the availability of sdAb sequence information enables these reagents to be produced by any researcher for evaluation and incorporation into their own research and development.

The coronavirus disease 2019 (COVID-19) pandemic, caused by the severe acute respiratory syndrome coronavirus-2 (SARS-CoV-2), highlighted the critical need for rapid, low cost, sensitive, and reliable diagnostic assays for emerging viral diseases. The ideal assay format would be rapid and multiplexed, enabling the processing of many samples and simultaneously providing information on the presence of known diseases, while also warning of a potential new emerging disease. High-throughput Luminex MagPlex immunoassays are relatively fast and simple, while providing the additional capability to be multiplexed. MagPlex assays use the sandwich format in which a capture reagent is immobilized on a color-coded magnetic MagPlex microsphere. After target binds to the bead-immobilized reagent, a biotinylated reporter reagent binds, and target binding is detected through streptavidin-phycoerythrin (SA-PE). The instrument reads both the identity of the MagPlex microsphere as well as the signal from the phycoerythrin. The Luminex MAGPIX instrument enables multiplexed and simultaneous detection of up to 50 targets per well using color-coded MagPlex beads (8).

The SARS-CoV-2 genome encodes four major structural proteins: spike (S), envelope (E), membrane (M), and nucleocapsid (N) proteins (9). These four main structural proteins are also found in other coronaviruses (10). The N protein of SARS-CoV-2 is an attractive target for diagnostic assays due to its abundance and its relative conservation (11, 12). The majority of commercially available antigen tests for COVID-19 are responsive to N (12). Currently most commercial tests for COVID-19 rely on conventional antibodies, however several groups, including our own, have developed sdAbs that target N (13–16).

We had previously developed a sdAb-based Luminex MagPlex immunoassay for the detection of N from SARS-CoV-2 (14). Our assay utilized a bivalent sdAb construct immobilized on the bead that was paired with a biotinylated bivalent sdAb construct that generated signal when exposed to SA-PE. In this report, we expand our previous work to include the oriented immobilization of the bivalent sdAb capture and testing of clinical samples.

## 2 Materials and methods

### 2.1 Materials

Unless otherwise specified, chemical reagents were from Sigma Aldrich (St. Louis, MO, USA), Thermo Fisher Scientific (Waltham, MA, USA), or VWR International (Radnor, PA, USA). Restriction endonucleases and ligation reagents were from New England Biolabs (Ipswich, MA, USA). Eurofins Genomics (Louisville, KY, USA) performed DNA sequencing and construction of gene fragments. Recombinant nucleocapsid from SARS-CoV-2 (N) expressed in HEK293 cells was from ACRO Biosystems (Newark, DE, USA). Recombinant N from SARS-CoV-2 (N) expressed in *E. Coli*, N from HKU1, OC43, NL63, 229E, and SARS-CoV were from the Native Antigen Company (Kidlington, UK). Middle East respiratory syndrome coronavirus (MERS-CoV) nucleoprotein was from Creative Diagnostics (Shirley, NY, USA). Magplex magnetic microspheres were from Luminex (Austin, TX, USA). Protein sequences of the sdAb constructs and SpyCatcher used in the work are provided in **Supplementary Table S1**.

The samples used in this study are post-residual clinical nasopharyngeal samples that were previously sent to and tested at the Naval Infectious Diseases Diagnostic Laboratory. The samples were de-identified prior to being used in the experiment. The samples had previously been tested for SARS-CoV-2 or other respiratory illnesses using TaqPath™ COVID-19 Combo Kit, (Thermo Fisher Scientific, MA, USA) CDC Influenza SARS-CoV-2 (Flu SC2) Multiplex Assay, or BioFire^®^ Respiratory Panel 2.1 (BioFire Diagnostics, UT, USA).

### 2.2 SdAb constructs and protein purification

Bivalent sdAb construct E2-C2 (14) was modified to include a C-terminal SpyTag sequence (17). The original E2-C2 construct encoded a NotI restriction enzyme cleavage site after the E2 component. To facilitate cloning into a pET22b vector containing the sequence for SpyTag between NotI and XhoI sites, a version of E2-C2 with flanking NcoI and NotI cleavage sites that contained no internal NotI site was synthesized as a gene fragment. The fragment was digested with the flanking enzymes, purified using a QIAquick PCR cleanup kit (Qiagen, Germantown, MD, USA), and ligated into the SpyTag containing vector that had been digested with the same enzymes, treated with phosphatase, and cleaned using the same QIAquick kit. The resulting construct was termed E2-C2-ST.

We employed the SpyCatcher 003 version of the SpyCatcher protein in conjunction with the original SpyTag sequence for this work (18). The sdAb constructs and SpyCatcher were produced using protocols identical or similar to those described previously (14, 19). Briefly, for preparation of SpyCatcher and the multivalent sdAb C2-B6, the Tuner™(DE3) strain of *E. coli* was used for expression, and cells were grown in terrific broth at 25°C, and induced for two hours. For preparation of the E2-C2-ST, induction was carried out overnight at 25°C. For all preparations, cells were pelleted and subjected to an osmotic shock process followed by immobilized metal affinity chromatography and fast protein liquid chromatography. Concentrations were determined by the absorbance at 280 nm using a Nanodrop. Preparations were aliquoted and stored frozen at -80°C until use. A more detailed protocol is provided in the **Supplementary Information**.

### 2.3 MagPlex assay

The E2-C2 sdAb construct and SpyCatcher were immobilized on unique sets of MagPlex microspheres (carboxylated magnetic beads) using 30 µL of each bead set and the standard immobilization protocol provided by the manufacturer. Using 1-Ethyl-3-(3-dimethylaminopropyl)carbodiimide (EDC) and sulfo-N-hydroxysulfosuccinimide (Sulfo-NHS), primary amines on the proteins were coupled to the carboxyl groups on the surface of the microsphere. Oriented capture microspheres were created by incubating beads conjugated with SpyCatcher with E2-C2-ST (50 µg) overnight. Unbound E2-C2-ST was removed by washing three times with PBST (PBS containing 0.05% Tween-20) prior to storage at 4^ο^C.

The reporter sdAb construct, C2-B6, was biotinylated as described previously (14), with Sulfo-NHS-LC-LC biotin. Briefly, about 300 µg of sdAb construct was used with a 10 to 1 molar ratio of Sulfo-NHS-LC-LC biotin to sdAb construct. Excess uncoupled biotin was removed using a Zeba spin column. The concentration of biotinylated sdAb was calculated using the absorbance at 280 nM.

Assays were similar to the amplified protocol we originally described for detecting N using MagPlex assays (14). All assay reagents were diluted into LowCross buffer (Candor, Wangen, Germany), and all washes were with PBST. Serial dilutions of N into LowCross buffer were used as the standard and they were prepared in a round-bottom polypropylene microtiter plate such that each well contained 90-100 µL of sample. Next, the sdAb-coated microspheres were added in a final volume of ~ 4 µL to provide a minimum of 50 microspheres for each set per well and incubated in the dark for 1 hour. Plates were washed two times with 200 µL of PBST using a 96f magnet (BioTek, Winooski, VT, USA) and then 50 µL biotinylated sdAb was added at 1 μg/mL for 30 minutes. To generate the fluorescent signal, the plate was washed and then incubated sequentially with 50 µL of SA–PE at 5 µg/mL in each well for 15 min, washed again, then incubated with 50 µL of biotinylated goat anti-streptavidin from Vector Laboratories (Burlingame, CA, USA) at 1 µg/mL for 15 min, washed, and finally incubated with SA–PE as before. Then, the plate was washed a final time prior to being evaluated on the MAGPIX. A similar protocol was followed for clinical samples. For those samples, beads were diluted in LowCross buffer to yield a minimum of 50 microspheres when added at 100 µL per well. Then 100 µL of recombinant N controls or clinical sample was added to wells. Further steps were as described above.

## 3 Results

Previously we had reported three sdAbs (E2, C2, and B6) that each bind to N from SARS-CoV-2 with nanomolar affinity (14). Sandwich immunoassays indicated that these clones bind distinct epitopes as they paired with each other, but exhibited competitive inhibition with themselves (14). This was confirmed by structural studies which showed that clone E2 recognized the C-terminal dimerization domain while clones C2 and B6 bind the N-terminal RNA binding domain (20). We developed a sensitive MagPlex-based immunoassay for the detection of N using bivalent constructs where pairs of these sdAbs were genetically linked through a flexible peptide linker; the best combinations included using the E2-C2 bivalent capture combined with the C2-B6 bivalent reporter (14).

The original assay format involved immobilizing the capture construct through conventional EDC chemistry which results in non-oriented captures, some of which may be unable to bind antigen due to their immobilization. In previous work, we demonstrated improved detection using capture constructs that were oriented on MagPlex beads to increase likelihood that the sdAb binding region was available to bind target (19). Orientation was accomplished through use of the SpyCatcher and SpyTag pair (17). In the SpyCatcher/SpyTag system, an irreversible covalent bond is spontaneously formed between the SpyTag peptide and SpyCatcher protein. We engineered the bivalent E2-C2 sdAb-based capture constructs to include a C-terminal SpyTag; the resulting construct was called E2-C2-ST. The SpyCatcher protein was immobilized onto MagPlex beads using EDC chemistry, and the E2-C2-ST capture construct was added to the SpyCatcher functionalized beads to form the oriented capture.

**Figure 1:**
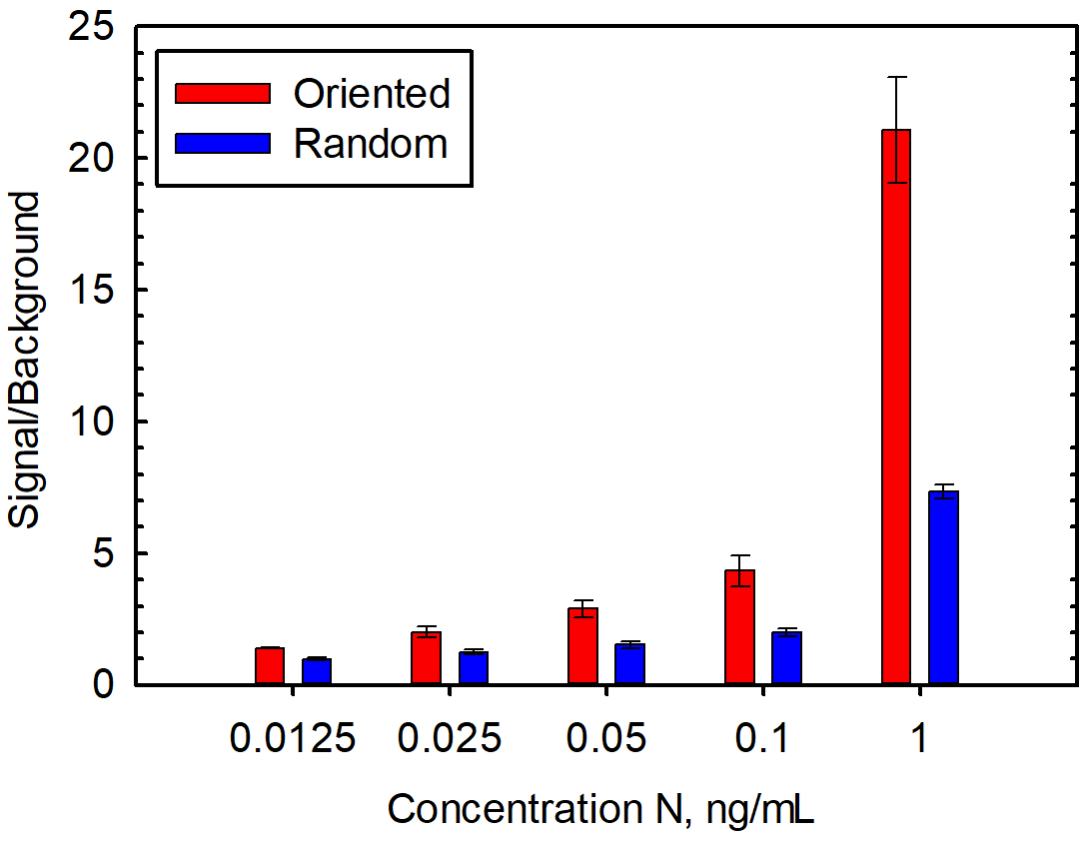
Detection of recombinant N spiked into buffer with oriented and random capture. Data is the average of five replicate experiments performed on separate days, and the error bars represent the standard error.

We hypothesized that using an oriented capture would improve N detection, thus providing a more sensitive assay for the evaluation of clinical samples. First, we examined the detection of recombinant N in buffer with both the oriented and random capture. Using the oriented capture, we reproducibly observed more sensitive detection of N versus the non-oriented version on performing numerous dose-response experiments that examined several different concentration ranges. **Figure 1** shows representative dose-response data using random and oriented capture. Limits of detection of these experiments were 25 pg/mL for the oriented capture and 100 pg/mL for the random capture. Importantly, two independently conjugated batches of oriented capture showed superior performance to two independently prepared batches of non-oriented capture beads suggesting that this result was not an artifact due to a batch-to-batch difference in beads.

**Figure 2:**
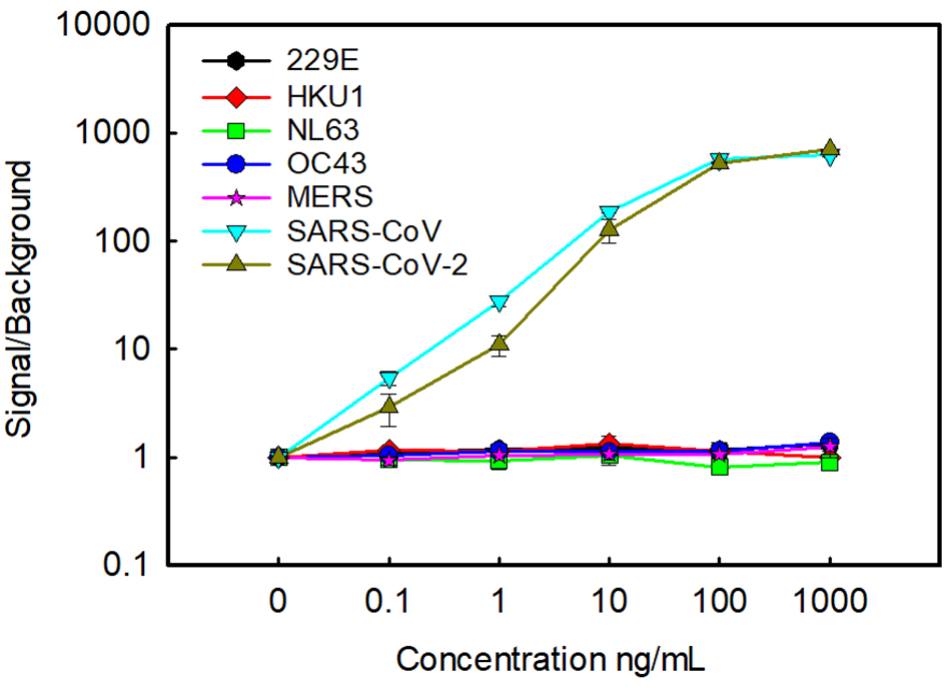
Cross reactivity with other coronaviruses. Oriented capture was used to examine the cross reactivity with N from seasonal human coronaviruses, MERS and SARS-CoV. For comparison detection of N from SARS-CoV-2 was also included. Data is the average of two replicate samples, error bars represent the standard error.

Using the oriented capture, we confirmed the specificity of the assay, examining recombinantly produced N from unrelated human seasonal coronaviruses as well as MERS-CoV and SARS-CoV (**Figure 2**). As we had seen previously, there was no cross reactivity with human seasonal coronaviruses HKU1, 229E, NL63, and OC43. No signal was seen with MERS-CoV either, however, consistent with our previous results, there is strong cross reactivity with SARS-CoV (14).

The next step was to determine the ability of the sdAb-based MagPlex assay to detect N in clinical samples. A summary of results from testing with clinical samples are compiled in **Table 1**.

**Table 1:** COVID-19 detection in clinical samples using oriented and random sdAb capture constructs.

	Ct value^[a]^	# Samples	Positive^[b]^	Negative^[c]^	Sensitivity %
Oriented	Ct <20	5	5	0	100
Oriented	20≤ Ct <25	23	18	5	78
Oriented	Ct >25	31	8	23	26
Random	Ct <20	5	5	0	100
Random	20≤ Ct <25	5	1	4	20
Random	Ct >25	12	0	12	0

[a] Previously determined. [b] Signal to background Ratio >1.5. [c] Signal to background Ratio <1.5.

We started with a group of 22 samples positive for COVID-19, four positive samples from each of the human seasonal coronaviruses HKU1, 229E, NL62, and OC43, and four negative samples. These respiratory samples had been previously tested by real-time reverse transcriptase PCR (RT-PCR), the standard laboratory method for the diagnosis of COVID-19. We chose samples positive for COVID-19 that spanned a range of threshold cycle (Ct) values from under 20 to over 32. The Ct value is inversely proportional to the amount of viral nucleic acid in a clinical sample and has been correlated with the concentration of N in the sample (21). We tested each clinical sample in duplicate using both the oriented and non-oriented capture, and a dilution series of recombinant N in buffer was run at the same time as the samples (**Supplementary Figure S1**). Background signal was defined as the value of control samples with buffer only. The eight readings from the negative samples had signal/background ratios ranging from 0.8 to 1 with an average of 0.9 and standard deviation of 0.1. We set the threshold for calling samples positive at a signal/background ratio of 1.5, which is greater than three standard deviations over the mean of the COVID-19 negative samples, and a value that yielded no false positives. All five of the high titer samples (Ct <20) gave a ratio over 2. Four of the oriented medium titer samples (20≤ Ct <25), had signal to background ratios of 1.8 or higher, yielding 80% sensitivity in this category. Whereas the randomly-oriented capture reagent performed well with the five high titer samples, it did not do as well as the oriented capture for the five samples with a medium titer, with only one sample in this group showing a signal to background ratio of over 1.5. The 12 low titer COVID-19 samples (Ct >25) all had an average signal/background ratio 1.1 or under for both oriented and random captures. These results indicate that, at the very least, using the oriented capture provided microspheres in which the binding paratopes of the E2-C2 sdAbs was less obscured, translating to higher signal when N is abundant. All of the 229E, NL62, and OC43 samples were negative. One of the HKU1 samples was co-infected with SARS-CoV-2 as assessed by RT-PCR and also was positive in our assay using both oriented and non-oriented capture, the other three HKU1 samples tested negative.

A second set of 42 clinical samples, including five negative samples and positive samples with Ct values ranging from 20 to 30, was examined using only oriented capture reagent. Each sample was run in duplicate (**Supplementary Figure S1**). The average and standard deviation from the 10 readings from the negative controls was 1.1 and 0.1 respectively; as before, a signal to background ratio of at least 1.5 was defined as positive as it was distinct from the negative control values and yielded no false positives. All the medium titer samples that tested positive, and four of the high titer samples had signal to background values of at least 2, with four additional high titer samples giving ratios of between 1.5 and 2. Results were consistent with the first set of results with 78% of the medium titer samples identified. Additionally, 42% of the low titer samples were identified as positive in the second set.

## 4 Discussion

Overall, our developed sdAb-based MagPlex immunoassay for SARS-CoV-2 N protein shows potential for diagnostic use. Although RT-PCR tests are still the gold standard for COVID-19 diagnostics, antigen tests have shown their value. RT-PCR tests are highly sensitive. It was shown that Ct values correlate with the ability to propagate virus from clinical samples and one study showed that viable virus was isolated in five of 60 samples with a Ct value over 35 (22). However, RT-PCR tests have the pitfall that they can still show positive results when patients are no longer infectious. Positive results with antigen tests, on the other hand, correlate better with higher viral loads that indicate transmissible virus (23).

The performance of our assay appears comparable to another study that integrated sdAbs into a diagnostic sandwich immunoassay in a plate-based format where a sdAb-luciferase fusion provided signal (15). In that study, as with ours, 100% sensitivity was observed with Ct values under 20, while detection when Ct values were over 20 were not as sensitive. Although Ct values are not standard and can vary due to factors such as sample collection method and the specific RT-PCR test used, both assays showed similar limits of detection using recombinantly produced N (~ 50 pg/mL), so it is reasonable to hypothesize they would have similar detection of clinical samples. Plate-based assays have the advantage of only requiring plate readers that are commonly found in laboratories, however, most do not have the potential for multiplexing that can be achieved with MagPlex beads and the MAGPIX instrument where up to 50 independent assays can be performed in each well.

Our assay is approaching the sensitivity of commercial rapid tests which provided reliable results in samples with Ct values under 25 (24). One of the benefits of sdAbs as recognition elements is their ability to be tailored for specific applications. Engineered oriented bivalent constructs outperformed randomly oriented ones. Potentially we could achieve fewer false negatives with low titer samples through the use of multimeric formats. This could include strategies such as adding a domain to produce pentamers of our bivalent constructs (25), or using orthogonal catcher/tag systems to produce oriented dimers or trimers of the bivalent capture construct (26). Another benefit of sdAbs is their ability to access hidden epitopes. It was observed that sdAbs can bind conserved regions on microbes and viruses (27); several studies have described sdAbs that have broad recognition within related viruses (28, 29). The MagPlex bead assay format enables the potential to integrate the sdAb-based COVID-19 test with assays for other respiratory viruses. Such an assay could be advantageous to patient care when multiple respiratory viruses are in circulation. It also has the potential to serve as a sentinel for the detection of novel and emerging viruses with the inclusion of recognition elements that bind conserved viral epitopes that enable identification of multiple related viruses.

## Data Availability

The original contributions presented in the study are included in the article/**Supplementary Material**. Further inquiries can be directed to the corresponding authors.

## Author disclaimer

SJ is a military Service member. This work was prepared as part of her official duties. Title 17, U.S.C., §105 provides that copyright protection under this title is not available for any work of the U.S. Government. Title 17, U.S.C., §101 defines a U.S. Government work as a work prepared by a military Service member or employee of the U.S. Government as part of that person’s official duties.” The views expressed in this article are those of the authors and do not necessarily reflect the official policy or position of the Department of the Navy, the Department of Defense, the US government, Uniformed Services University or the Henry M. Jackson Foundation for the Advancement of Military Medicine, Inc.
