# Supplementary material for: A single domain antibody-based Luminex assay for the detection of SARS-CoV-2 in clinical samples
Source: Front Immunol. 2024 Aug 13;15:1446095. doi: 10.3389/fimmu.2024.1446095 (PMC11347438; doi:10.3389/fimmu.2024.1446095)
Supplement: Supplementary file 1 [file DataSheet1.pdf]

Supplementary Material

A Single Domain Antibody-based Luminex Assay for the Detection of SARS-CoV-2 in Clinical Samples

Ellen R. Goldman[1*], Victor A. Sugiharto[2,3], Lisa C. Shriver-Lake[1], Andrew M Garcia[2,4], Shuenn-Jue Wu[2], Sarah A Jenkins[2], Hua-Wei Chen[2,3*]

[1]Center for Biomolecular Science and Engineering, US Naval Research Laboratory, 4555 Overlook Ave SW, Washington, DC 20375, USA

[2]Diagnostic and Surveillance Department, Naval Medical Research Command, 503 Robert Grant Avenue, Silver Spring, MD 20910, USA

[3]Henry M Jackson Foundation, Bethesda, MD 20817, USA

[4]Leidos Inc., Reston, VA 20190, USA

*** Correspondence:**
Ellen R. Goldman
ellen.r.goldman.civ@us.navy.mil
Hua-Wei Chen
huawei.w.chen.ctr@health.mil

Table S1: Protein sequences of sdAb constructs and SpyCatcher 003 used in this work

Protein	Amino Acid sequence (single letter code)	reference[a]
E2-C2	EVQLQASGGGLVQAGGSLRLSCAASGRTDSTQHMAWFRQAPGKER<br>EFVTAIQWRGGGTSYTDSVKGRFTISRDNAKNTVYLEMNSLKPED<br>TAVYYCATNTRWTYFSPTVPDRYDYWGQGTQVTVSSAAAGGGGSG<br>GGGSGGGGSGSEVQLQASGGGLVRPGGSLRLSCAASGFTFSSYAM<br>MWVRQAPGKGLEWVSAINGGGGSTSYADSVKGRFTISRDNAKNTL<br>YLQMNSLKPEDTAVYYCAKYQAAVHQEKEDYWGQGTQVTVSS	14
E2-C2-ST	EVQLQASGGGLVQAGGSLRLSCAASGRTDSTQHMAWFRQAPGKER<br>EFVTAIQWRGGGTSYTDSVKGRFTISRDNAKNTVYLEMNSLKPED<br>TAVYYCATNTRWTYFSPTVPDRYDYWGQGTQVTVSSAAAGGGGSG<br>GGGSGGGGSGSEVQLQASGGGLVRPGGSLRLSCAASGFTFSSYAM<br>MWVRQAPGKGLEWVSAINGGGGSTSYADSVKGRFTISRDNAKNTL<br>YLQMNSLKPEDTAVYYCAKYQAAVHQEKEDYWGQGTQVTVSSAAA<br>GGGGSGGGGSGSAHIVMVDAYKPTKGSGLE	This work
C2-B6	EVQLQASGGGLVRPGGSLRLSCAASGFTFSSYAMMWVRQAPGKGL<br>EWVSAINGGGGSTSYADSVKGRFTISRDNAKNTLYLQMNSLKPED<br>TAVYYCAKYQAAVHQEKEDYWGQGTQVTVSSAAAGGGGSGGGGSG<br>GGGSGSEVQLQASGGGLVQAGDSLRLSCVAVSGRTISTFAMGWFR<br>QAPGKEREFVATINWSGSSARYADPVEGRFTISRDDAKNTVYLEM<br>SSLKPGDSAVYYCASGRYLGGITSYSQGDFAPWGQGTQVTVSS	14
SpyCatcher 003	VTTLSGLSGEQGPSGDMTTEEDSATHIKFSKRDEDGRELAGATME<br>LRDSSGKTISTWISDGHVKDFYLYPGKYTFVETAAPDGYEVATPI<br>EFTVNEDGQVTVDGEATEGDAHT	18

[a] Refers to the reference numbers from the manuscript

14. Anderson GP, et al. Single-Domain Antibodies for the Detection of SARS-CoV-2 Nucleocapsid Protein. Anal Chem. 2021;93(19):7283-91. doi: 10.1021/acs.analchem.1c00677

18. Keeble AH, et al. Approaching infinite affinity through engineering of peptide–protein interaction. Proceedings of the National Academy of Sciences. 2019;116(52):26523-33. doi: 10.1073/pnas.1909653116

Figure S1

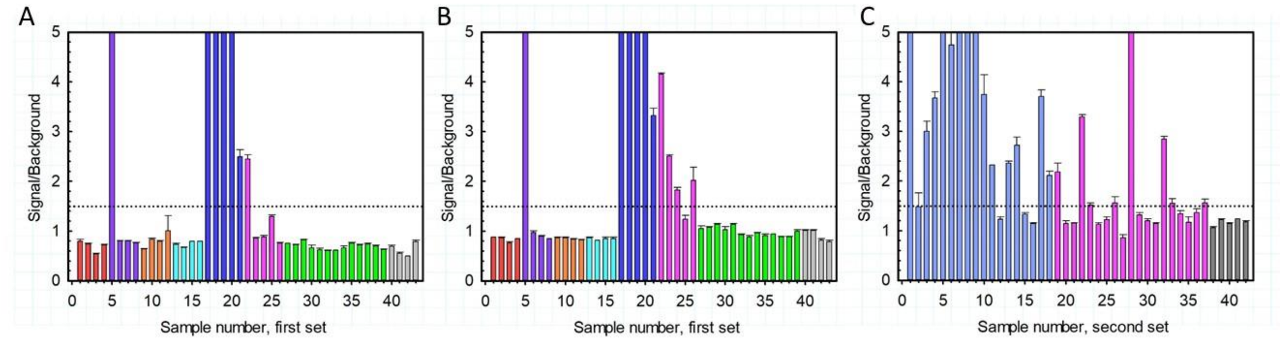

Results from clinical samples. Panel A shows randomly oriented capture, while B and C are oriented capture. Within the first set of samples (panels A and B), sample numbers 1-4 are 229E, 5-8 are HKU1, 9-12 are NL63, 13-16 are OC43, and 40-43 are negative samples. Samples 17-39 are COVID-19 positive by RT-PCR and are listed in increasing Ct with high titer samples 17-21, medium titer 22-26, and low titer 27-39. The second set of samples (C), is listed by increasing Ct with medium titer samples numbers 1-18, and low titer 19-38, Samples 39-42 are negative

Protein production protocol

Expression plasmids were transformed into Tuner (DE3) for protein production. Cultures were started from freshly transformed colonies inoculated into 50 mL of terrific broth (TB) containing 100 µg/mL ampicillin and grown at 25 °C overnight. The next day the overnight culture was poured into 450 mL TB (100 µg/mL ampicillin). For the E2-C2, C2-B6, and SpyCatcher003 preparations, cultures were grown for 2 h at 25 °C, induced by the addition of IPTG (final concentration of 0.5 mM), and grown an additional 2 h at 25 °C. The E2-C2-ST culture was grown 6-8 hours further at 25 °C prior to inducing overnight with IPTG (final concentration of 0.5 mM).

After induction, cells were centrifuged and pelleted cells from each 500 mL shake flask culture were resuspended in 14 mL of Tris-Sucrose buffer (100 mM Tris, 0.75M sucrose pH 7.5) by gently smushing them with a spatula. Next 1 mL of lysozyme (1 mg/mL) made up in the Tris-Sucrose buffer was added to the homogenized cells followed by 28 mL of 1 mM EDTA added dropwise to the solution while the centrifuge tubes were held in crushed ice shaking on a rotating platform. After addition of the EDTA, 0.25 mL of 5% deoxycholate was added and the cells were gently swirled for another half hour. Lastly, 1 mL of 0.5M $MgCl_2$ was added and the mix incubated for another 15 minutes, prior to pelleting the spheroplasts. The supernatant was poured into a 50 mL conical tube that contained 5 mL of 10× IMAC buffer (0.2 M Na_2HPO_4, 4 M NaCl, 0.2 M imidazole, pH 7.5) and 0.5 mL of Ni Sepharose (GE Healthcare) that had been washed in 1X IMAC buffer. The sample tumbled at least 1 h at 4 °C on a rotisserie. Next, the resin was washed twice in batch with 25 mL 1× IMAC buffer. The resin was poured into a small column, washed with a further ~10 mL 1× IMAC buffer and eluted with 1 mL of 1× IMAC buffer containing 250 mM imidazole. Protein was then further purified into PBS by size exclusion chromatography using a Bio-Rad Enrich SEC70 10 300 column (or a SEC 650 10x 300 column depending on the size of the construct) and a Bio-Rad Duo-Flow System. Yield of the sdAbs was determined by UV spectroscopy using a Nanodrop (Thermo).
